# Age, serum 25-hydroxyvitamin D and vitamin D receptor (VDR) expression and function in peripheral blood mononuclear cells

**DOI:** 10.18632/oncotarget.9398

**Published:** 2016-05-17

**Authors:** Laura A. Coleman, Margarita Mishina, Mark Thompson, Sarah M. Spencer, Adrian J. Reber, William G. Davis, Po-Yung Cheng, Edward A. Belongia, H. Keipp Talbot, Maria E. Sundaram, Marie R. Griffin, David K. Shay, Suryaprakash Sambhara

**Affiliations:** ^1^ Abbott Nutrition, Columbus, OH, USA; ^2^ Battelle, Columbus, OH, USA; ^3^ U.S. Centers for Disease Control and Prevention, Atlanta, GA, USA; ^4^ Marshfield Clinic, Marshfield, WI, USA; ^5^ Vanderbilt University, Nashville, TN, USA; ^6^ University of Minnesota School of Public Health, Minneapolis, MN, USA

**Keywords:** vitamin D, vitamin D receptor, 1α-hydroxylase, mRNA expression, PBMC, Gerotarget

## Abstract

The relationship between age, vitamin D status, expression and functionality of the vitamin D receptor (VDR), and key genes in the vitamin D pathway in immune cells is unclear. We enrolled adults 50 to 69 years old (20 subjects) and 70+ (20 subjects) and measured: 1) 25(OH)D levels by liquid chromatography/mass spectrometry; and 2) mRNA expression of VDR, 1α-OHase, 1,25D_3_-MARRS, TREM-1, cathelicidin, RIG-I, and interferon-β by qRT-PCR. Mean serum 25(OH)D was 30 ± 4 ng/mL and was not associated with age. Baseline expression of VDR, 1α-OHase, 1,25D_3_-MARRS, TREM-1, and RIG-I also did not differ by age; IFN-β expression, however, was higher in the 70+ year old group. 25(OH)D_3_- and 1,25(OH)_2_D_3_-induced VDR, TREM-1 and cathelicidin expression were similar between age groups, as was LPS-induced expression of VDR and of 1α-OHase. Ligand-induced 1,25D_3_-MARRS expression was higher in subjects ≥ 70 years. Serum 25(OH)D was inversely associated with LPS-stimulated VDR expression and with baseline or vitamin D-induced TREM-1 expression, adjusting for age, self-rated health, and functional status. In healthy adults ≥ 50 years, the expression and functionality of the VDR, 1α-OHase and key vitamin D pathway genes were not consistently associated with age.

## INTRODUCTION

Interest in the extra-skeletal effects of vitamin D has grown rapidly over the past thirty years with the identification of vitamin D receptors (VDRs) in various organ systems and cell types throughout the body [1, 2]. The biologically active form of vitamin D, 1,25(OH)_2_D_3_, is produced by two hydroxylation reactions, the final one of which occurs mostly in the kidney via 1α-hydroxylase (1α-OHase). Binding of the active form of vitamin D to its receptor leads to heterodimerization of the VDR with the retinoid-x receptor; the resulting complex binds to responsive elements in DNA and regulates the expression of several gene products involved in calcium and phosphorous absorption and metabolism, skeletal muscle function, bone metabolism, parathyroid function, and regulation of inflammation [2].

The effects of 1,25(OH)_2_D_3_ on the regulation of both the innate and adaptive immune systems are extensive, and are only beginning to be appreciated. The VDR has been found in activated CD4+ and CD8+ T cells, B cells, neutrophils, monocytes, macrophages, and dendritic cells [3]. Binding of vitamin D to VDR in macrophages, neutrophils and monocytes leads to the secretion of the antibacterial peptide, cathelicidin, which plays an important role in innate immune defenses via its ability to lyse bacteria [4]. Serum 25(OH)D levels have been associated with expression and functionality of certain toll like receptors (TLRs), especially those involved in viral responses [5]. Activation of innate immune receptors, such as TLR2, enhances the expression of VDR, 1α-OHase and cathelicidin, suggesting a potential role for vitamin D in innate immune responses against bacterial pathogens [6]. Vitamin D has also been shown to inhibit T cell expansion and modulate the expression of cytokines with a Th2 bias [7]. Vitamin D inhibits differentiation and proliferation of B-lymphocytes, class switching and immunoglobulin secretion. Other immune effects that have been attributed to vitamin D include maturation of dendritic cells, down-regulation of class II MHC expression and enhancement of antigen processing and presentation, leading to the induction of more tolerogenic cytokines such as IL-10 [3, 8]. This altered priming environment influences Th cell differentiation into Th2 [9, 10].

Potential clinical implications of the presence of VDR in immune cells include a possible role in autoimmunity, infectious diseases and cancer. For example, an association between vitamin D deficiency and the increased incidence of autoimmune diseases, namely, systemic lupus erythematosus, rheumatoid arthritis, multiple sclerosis, and inflammatory bowel disease in humans, and increased incidence of autoimmune disorders in animal models deficient in vitamin D function, have been reported and reviewed recently by Prietl et al. [11]. Vitamin D has been shown to inhibit the growth of mycobacterium tuberculosis in human macrophages through the secretion of antibacterial peptides [12]. Recent studies have also suggested that vitamin D has a beneficial effect in terms of patient survival in non-small cell lung cancer [13] and may act as a VDR agonist and therapeutic agent in *EGFR* mutant lung cancer [14].

Evidence has accumulated that 1,25(OH)_2_D_3_ regulates cell processes not only by traditional nuclear receptor-mediated transcriptional regulation (via VDR) but also by rapid signal transduction via the membrane receptor 1,25D_3_-MARRS (Membrane Associated Rapid Response Steroid-binding) [15, 16]. There is also evidence that VDR plays a role in non-transcriptional plasma membrane initiated signaling, and that 1,25D_3_-MARRS- NFĸB translocation into the nucleus may play a role in differentiation of the NB4 cell line along the monocyte/macrophage lineage [17, 18]. Data on the physiological role of membrane-initiated action of 1,25(OH)_2_D_3_ are limited, but mechanisms may involve 1,25(OH)_2_D_3_ -mediated signal transduction in cell proliferation as an early step in growth inhibition, likely followed by VDR-mediated transcriptional regulation of proliferation [19]. Cross-talk between the two modes of vitamin D signaling may occur via targeted phosphorylation of critical proteins in the VDR-containing transcriptional complex [17, 20]. For example, antagonistic functions of 1,25D_3_-MARRS and VDR have been observed in breast cancer cells [18].

Altered physiological functions resulting in a dysregulated immune response to infectious diseases and enhanced susceptibility are a hallmark of aging [21, 22]; this dysregulated immune status is referred to as immunosenescence [23, 24]. During aging, there is an increased incidence of colonization of bacteria and fungi on epithelial and mucosal surfaces, reactivation of latent and chronic infections and increased susceptibility to infectious diseases [25, 26]. In addition, the immunogenicity and efficacy of preventive vaccines against bacterial and viral targets decline with aging [27, 28]. We and others have shown that the expression and function of innate immune receptors on macrophages and dendritic cells decline with aging [29–31]. Furthermore, we have also shown that reduced function of antigen presenting cells contributes to immune dysfunction in aging, which can be restored by either providing co-stimulation at the time of vaccination or formulating vaccines with adjuvants [31–34].

Since circulating levels of biologically inactive 25(OH)D need to be converted into the active form in order to have functional consequences, the expression and function of VDR, 1,25D_3_-MARRS as well as 1α-OHase influence downstream effects. As information is limited on the expression and function of VDR, 1,25D_3_-MARRS and 1α-OHasein aging, we investigated the association between age and the expression and function of VDR, 1,25D_3_-MARRS and 1α-OHase in peripheral blood mononuclear cells (PBMCs) in healthy vitamin D replete adults ≥ 50 years old. To determine the functionality of VDR, we measured the expression of human antibacterial peptide cathelicidin; triggering receptor expressed on myeloid cells 1 (TREM-1), a receptor of the innate immune system which is known to be induced by vitamin D [35]; retinoic acid inducible gene (RIG)-I and interferon (IFN)-β genes, which play an important role in the response to viral challenges including influenza [36].

## RESULTS

### Characteristics of the study participants

**Figure 1:**
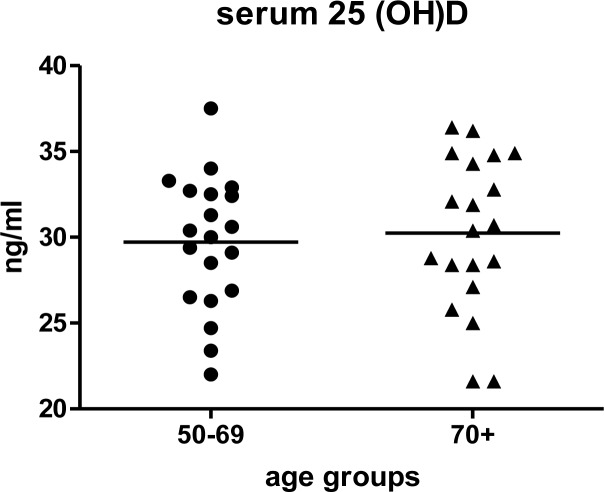
Serum 25(OH)D by age group The participants were chosen with serum 25(OH)D levels in the sufficient range (mean ± SD, 30.0 ± 4.1. ng/mL) [48]. Serum 25(OH)D was similar for both age groups. The mean of the values within age groups is represented by the horizontal line.

Participant characteristics are shown in Table 1. The mean ± SD age of subjects was 69.8 ± 11.4 years old; 55% were female. Participants were, by study design, community-dwelling and ambulatory. Age and health status were directly correlated among study participants (Table 2), however, most participants (27/40, 68%) described themselves as being in very good or excellent overall health, and only 7/40 (18%) participants had elevated Vulnerable Elders Survey (VES-13) [37]scores (≥ 3) indicative of functional decline. As expected, participants aged ≥ 70 years had modestly higher VES-13 scores. Serum vitamin D levels were similar among those aged 50-69 and ≥ 70 years (29.7±3.9 and 30.2±4.5 ng/mL, respectively) (Figure 1).

**Table 1:** Participant characteristics

Characteristics	Scale^1^	Full Sample (*n* = 40)	Age 50-69 (*n* = 20)	Age 70+ (*n* = 20)	*P*-value between groups^3^
Age	Years	69.8 (11.4)	59.4 (4.5)	80.1 (4.8)	< 0.001
Female sex	N (col %)	22 (55)	10 (50)	12 (60)	NS
BMI	kg/m^2^	26.8 (5.0)	26.4 (5.2)	27.2 (5.0)	NS
Self-rated health	1=poor to 5=excellent	3.9 (0.9)	4.2 (0.9)	3.7 (0.9)	NS
VES-13^2^	0-10	1.2 (1.7)	0.3 (0.8)	2.1 (1.9)	<0.001
Number difficult daily activities from VES-13	Of 11 activities of daily living, number difficult to do (0-6)	1.2 (1.7)	0.7 (1.3)	1.8 (1.9)	<0.05
Serum 25(OH)D	ng/mL	30 (4.1)	29.7 (3.9)	30.2 (4.5)	NS

1 Mean (SD) unless otherwise noted

2 Vulnerable Elders Survey

3 Age-group comparisons were made using Student's T-test, with the exception of Chi-square for comparison of male and female participants.

**Table 2:** Correlations between age and other characteristics and gene expression

Characteristics/gene expression	Correlation (r) with age (years)
Female sex	0.18
VES-13 score	0.66*
Self-rated health	−0.36*
Serum 25(OH)D	−0.06
VDR	
Baseline	−0.23
1,25(OH)2D3-induced	−0.20
25(OH)D3-induced	−0.15
LPS-induced	−0.01
1α-OHase	
Baseline	0.28
1,25(OH)2D3-induced	0.04
25(OH)D3-induced	0.20
LPS-induced	0.15
TREM-1	
Baseline	−0.17
1,25(OH)2D3-induced	−0.18
25(OH)D3-induced	−0.22
LPS-induced	−0.14
IFN-β	
Baseline	0.32*
LPS-induced	0.45*
1,25D3-MARRS^1^	
Baseline	−0.14
1,25(OH)2D3-induced	0.26
25(OH)D3-induced	0.19
LPS-induced	0.46*

* *p* < 0.05

1 Assays completed on 21 of 40 subjects due to limited RNA.

### Baseline expression and functionality of VDR and 1α-OHase

**Figure 2:**
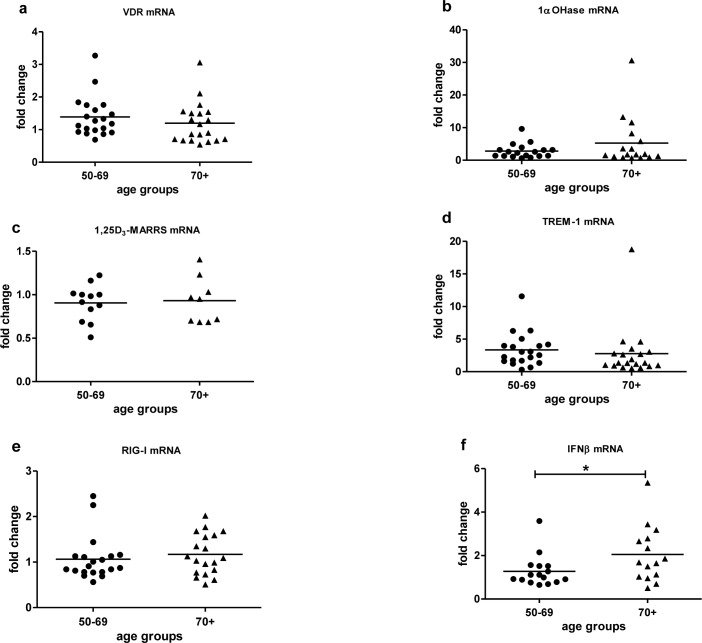
Baseline mRNA expression by age VDR **a.**, 1α-OHase **b.**, 1,25D_3_-MARRS **c.**, TREM-1 **d.**, RIG-I **e.**, IFN-β **f.** mRNA expression by age group was measured in untreated PBMCs and compared to expression in a calibrator sample (untreated random PBMCs). Gene expression was assessed by RT-qPCR. All assays were done in duplicate and expression was normalized to β-actin. The relative amount of target gene in each sample was estimated using the 2^−ΔΔCT^ method as described elsewhere [49]. The mean of the values is represented by the horizontal line. Statistical analysis was performed by Student's test (* *p* <0.05).

**Figure 3:**
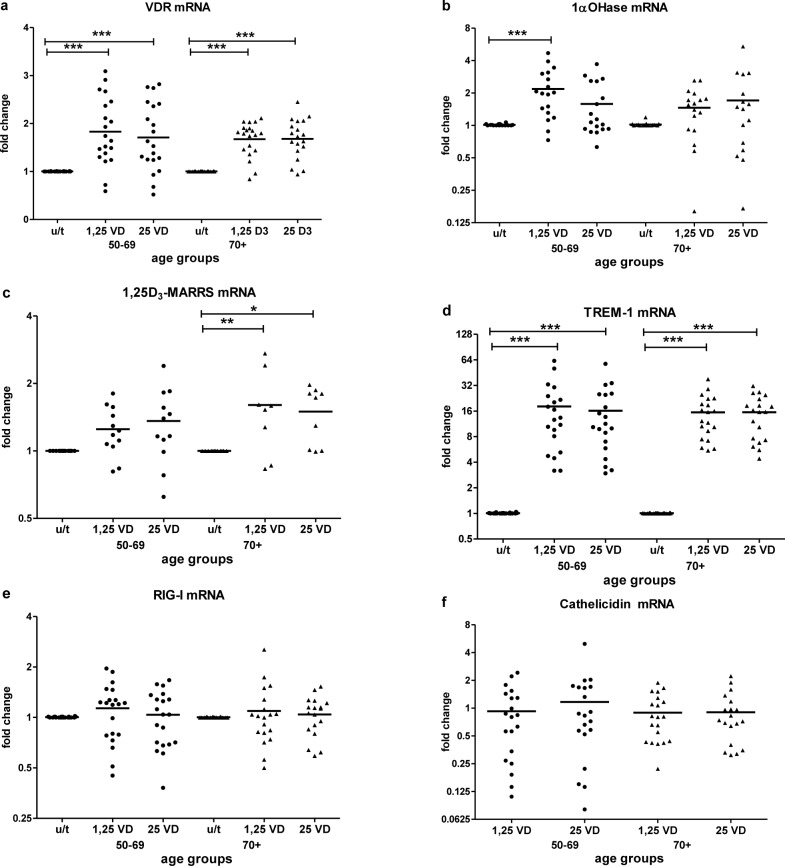
Vitamin D-induced mRNA expression by age The response to vitamin D stimulation by age group was determined by fold change in VDR **a.**, 1aOHase **b.**, 1,25D_3_-MARRS **c.**, TREM-1 **d.** and RIG-I **e.** mRNA expression after *in vitro* treatment of PBMCs with active 1,25(OH)_2_D_3_ or inactive 25(OH)D_3_ compared to untreated PBMCs. Cathelicidin **f.** mRNA expression was measured in vitamin D-treated samples and compared to expression in a calibrator sample (vitamin D treated random PBMCs) due to Cathelicidin mRNA at baseline was below the limit of detection. Gene expression was assessed by RT-qPCR. All assays were done in duplicate and expression was normalized to β-actin. The relative amount of target gene in each sample was estimated using the 2^−ΔΔCT^ method as described elsewhere [49]. The mean of the values is represented by the horizontal line.u/t: untreated PBMCs; 1,25 VD: PBMCs treated with 1,25(OH)_2_D_3_; 25VD: PBMCs treated with 25 (OH)D_3._ Statistical analysis was performed by ANOVA (* *p* < 0.05**, *p* < 0.01, *** *p* < 0.001).

Baseline expression of key vitamin D pathway genes, VDR (Figure 2a), 1α-OHase (Figure 2b) and 1,25D_3_-MARRS (Figure 2c), and of TREM-1 (Figure 2d) and RIG-I (Figure 2e) did not differ by age group (Table 2). Cathelicidin mRNA at baseline was below the limit of detection for all age groups. IFN-β mRNA expression was higher in subjects ≥ 70 *vs*. < 70 years (*p* < 0.05) (Figure 2f). *In vitro* exposure of PBMCs to either form of vitamin D - 1,25(OH)_2_D_3_ (the biologically active ligand) or 25(OH)D_3_ - induced VDR similarly in both age groups (Figure 3a). 1α-OHase was induced by vitamin D; this finding reached statistical significance only in the case of 1,25 (OH)_2_D_3_ treatment for subjects < 70 years (p≤0.001) (Figure 3b). 1,25D_3_-MARRS was induced by both vitamin D metabolites, but only reached significance for subjects ≥ 70 years (Figure 3c). As expected, either form of vitamin D induced TREM-1(*p* < 0.001) (Figure 3d) and cathelicidin expression (Figure 3f). The levels of induction were similar for both age groups (Table 2). Neither RIG-I (Figure 3e) nor IFN-β (data not shown)was affected by treatment of cells with the vitamin D metabolites.

**Figure 4:**
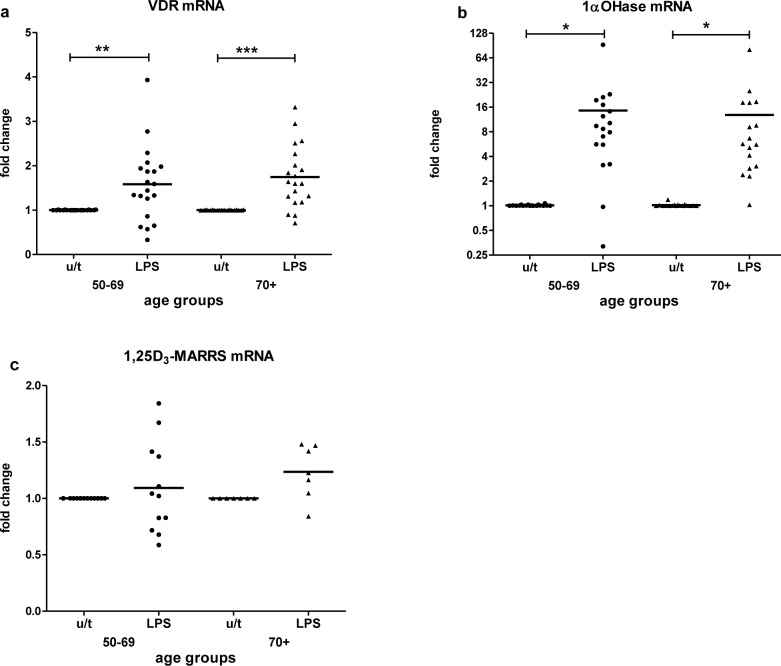
LPS-stimulated mRNA expression by age The response to LPS stimulation by age group was determined by fold change in VDR **a.**, 1α-OHase **b.** and 1,25D_3_-MARRS **c.** mRNA expression after *in vitro* treatment of PBMCs compared to untreated PBMCs. Gene expression was assessed by RT-qPCR. All assays were done in duplicate and expression was normalized to β-actin. The relative amount of target gene in each sample was estimated using the 2^−ΔΔCT^ method as described elsewhere [49]. The mean of the values is represented by the horizontal line. u/t: untreated PBMCs; LPS: PBMCs treated with LPS. Statistical analysis was performed by ANOVA (* *p* < 0.05, **p < 0.01, *** *p* < 0.001).

Assessment of VDR, 1α-OHase and 1,25D_3_-MARRS responsiveness to an immune stimulus was done by treating PBMCs with LPS. LPS upregulated the expression of VDR (*p* < 0.01) (Figure 4a) and 1α-OHase (*p* < 0.05) (Figure 4b) in both age groups equally. 1,25D_3-_MARRS was upregulated by LPS slightly for group 70+, not reaching statistical significance (Figure 4c); LPS-induced 1,25D_3_-MARRS expression was positively associated with age (Table 2).

### Correlation of gene expression and serum vitamin D concentration

We analyzed the association between VDR pathway/downstream vitamin D target genes and serum vitamin D concentrations (Table 3). LPS-stimulated VDR expression (r = −0.33) and baseline TREM-1 (r = −0.31) and vitamin D-stimulated (r = −0.40) TREM-1 expression were inversely associated with serum 25(OH)D, adjusting for age, self-rated health, and functional status (*p* < 0.05) (Table 3). Serum 25(OH)D concentration was not associated with 1,25D_3_-MARRS or 1α-OHase expression, or with IFN-β expression (Table 3).

**Table 3:** Association between serum 25(OH)D level and gene expression

Gene expression	Associations (Beta Coefficients) with Serum 25(OH)D^1^	
	Unadjusted	Adjusted
VDR		
Baseline	−0.07	−0.09
1,25(OH)_2_D_3_-induced	−0.29	−0.31
25(OH)D_3_-induced	−0.23	−0.25
LPS-induced	−0.32*	−0.33*
1α-OHase^2^		
Baseline	−0.04	−0.06
1,25(OH)_2_D_3_-induced	−0.25	−0.23
25(OH)D_3_-induced	−0.09	−0.07
LPS-induced	−0.20	−0.20
TREM-1		
Baseline	−0.31*	−0.31*
1,25(OH)_2_D_3_-induced	−0.38*	−0.40*
25(OH)D_3_-induced	−0.33*	−0.35*
LPS-induced	−0.39*	−0.41*
IFN-β^3^		
Baseline	0.07	0.06
1,25(OH)_2_D_3_-induced	−0.09	−0.08
25(OH)D_3_-induced	−0.08	−0.10
LPS-induced	−0.13	−0.12
1,25D_3_-MARRS^4^		
Baseline	−0.06	−0.08
1,25(OH)_2_D_3_-induced	−0.22	−0.36
25(OH)D_3_-induced	−0.28	−0.29
LPS-induced	−0.18	−0.25

* p < 0.05

1 Unadjusted associations are standardized Betas (correlations); adjusted for age, self-rated health, and sum of difficult daily activities from VES-13.

2 For stimulated conditions, assay completed on 35 of 40 subjects due to limited RNA.

3 Assay completed on 31 of 40 subjects due to limited RNA.

4 Assays completed on 21 of 40 subjects due to limited RNA.

## DISCUSSION

In this study of healthy, community-dwelling, vitamin D-replete adults 50 years and older, baseline level and functionality (responsiveness to vitamin D and LPS) of the VDR and 1α-OHase were not associated with age. LPS-induced 1,25D_3_-MARRS expression was positively associated with age. Serum 25(OH)D was within the normal range (30 ± 4 ng/mL) by study design, and there was an inverse association of LPS-induced VDR mRNA level and serum vitamin D. The downstream target gene TREM-1 (baseline) and vitamin D-induced mRNA expression were also inversely associated with serum vitamin D. These results suggest differential regulation of the vitamin D pathway in immune cells by serum vitamin D.

Bischoff-Ferrari et al. reported that older age was associated with decreased VDR protein expression in human skeletal muscle tissue, independent of serum 25(OH)D levels [38]. The disagreement with the current study may be because regulation of expression is tissue-specific. Moreover, their study included adults ranging from age 24 to 91 years old, contrary to our study which only included adults 50 years and older. Also, our older adult population was generally healthy; by study design, all subjects were free-living. Thus, this group of subjects likely does not represent an elderly population with advanced immune senescence and results cannot be generalized to all elderly individuals. Indeed, the participants in the current study may represent individuals with a longer “health span” rather than only a longer “life span” [39]. In addition, Bischoff-Ferrari et al. measured protein levels, while our study focused on mRNA expression. Post-transcriptional regulation of expression can be affected by aging. There have not been any reports on 1α-OHase and 1,25D_3_-MARRS expression being altered with aging.

In the present study, the expression of IFN-β, an important antiviral regulator of immunity, was higher in subjects over 80 years. This finding is in agreement with the previous reports that low-level chronic inflammation, or “inflammaging”, is commonly observed in older populations [40, 41]. IFN-β is regulated by multiple signaling pathways, with the RIG-I pathway being one of them [42]. However, we observed no regulation of IFN-β/RIG-I mRNA expression by vitamin D treatment of immune cells or association of IFN-β expression with circulating 25(OH)D.

Besides cellular VDR content and active ligand accessibility to the VDR, the biological VDR-mediated response to 1,25(OH)_2_D_3_ is also influenced by availability and activation status of nuclear co-regulators [43]. Information on the role of 1,25D_3_-MARRS in immune responses, including cross-talk with the VDR, is very limited, and all components of the vitamin D pathway in immune cells can be affected by age. Future studies are needed in a population with a range of vitamin D status and age, to address how vitamin D level and aging affect the VDR- 1,25D_3_-MARRS pathways in immune cells, both independently and synergistically.

## MATERIALS AND METHODS

### Study participants and data collection

As part of a larger study designed to investigate immune response to influenza vaccination, forty adults ≥ 50 years old (mean ± SD 69.8 ± 11.4 years) were enrolled at two sites, Vanderbilt University Medical Center (Nashville, TN) and Marshfield Clinic Research Foundation (Marshfield, WI), during the months of September through October 2008. The details of recruitment, data collection and blood collection/ processing have been described in detail elsewhere [44, 45]. To describe the risk of functional decline or death, participants completed the 13-item VES-13 [37], which results in a 10-point score with scores ≥ 3 associated with higher risk of death or functional decline. This measure presents 11 activities of daily living; we report the number (0 to 6 or more) described as difficult on average to complete (e.g., ability to stoop or crouch, to lift or carry heavy objects, perform heavy housework). The difficulty of 6 of these activities were rated as “no difficulty” (0) or “a little difficult” (1) to “unable to do” (4) and summed to form a difficulty score of 0 to 18 or higher. We also report on the participant's self-rated overall health status (1 = poor to 5 = excellent) [46, 47].

Blood samples from 20 participants in each of the following age groups were analyzed: 50-69 and ≥ 70 years. In order to minimize the influence of circulating 25(OH)D concentration on the vitamin D pathways in cells of the immune system, the participants chosen for inclusion in the present study had serum 25(OH)D levels of 20-40 ng/mL (based on the Institute of Medicine's definition of sufficiency) (Figure 1) [48]. Study procedures, informed consent documents and data collection forms were reviewed and approved by Institutional Review Boards at each of the study sites and Centers for Disease Control and Prevention.

### Laboratory methods

Serum 25(OH)Dwas measured by liquid chromatography/mass spectrometry [44]. 1×10^6^ PBMCs were stimulated with 200 ng/mL of 1,25(OH)_2_D_3_ or 25(OH)D_3_ or 100 ng/mL of LPS obtained from Sigma (St. Louis, MO), for 20 hrs. mRNA was purified using RNeasy mini kit (QIAGEN, Valencia, CA). Expression of VDR, 1,25D_3_-MARRS, 1α-OHase, TREM-1, cathelicidin, RIG-I, and IFN-β was measured by Real-Time quantitative Reverse Transcription Polymerase Chain Reaction (qRT-PCR) with SuperScript III Platinum SYBR Green One-Step qRT-PCR- kit (Life Technologies, Grand Island, NY) or with Transcriptor Reverse Transcriptase (Roche, Indianapolis, IN) followed by SYBR Green JumpStart Taq ReadyMix (Sigma, St. Louis, MO) with gene-specific primers (Table S1) using real-time detection instrument Mx3000P (Stratagene, Santa Clara, CA). All assays were done in duplicate and expression was normalized to β-actin. The relative amount of target gene in each sample was estimated using the 2^−ΔΔCT^ method as described elsewhere [49].

### Statistical analysis

Chi-square tests and Student's *T*-tests were used to compare the characteristics of participants by age group. We used linear regression models to describe the association between serum 25(OH)D levels and gene expression. Given that all study variables are likely associated with age and underlying health and functional status, we present standardized Beta coefficients adjusted for age, self-rated health status, and functional status (i.e., the sum of difficult daily activities).

## SUPPLEMENTARY MATERIALS TABLE



## Abbreviations

The following abbreviations are used in this manuscript:

25-hydroxyvitamin D: 25(OH)D
VDR: vitamin D receptor
1,25D_3_-MARRS: membrane associated, rapid response, steroid-binding receptor protein
PBMC: peripheral blood mononuclear cells
1,25(OH)_2_D_3_: 1, 25 dihydroxyvitamin D_3_
1α-OHase: 1 alpha hydroxylase
TLR: toll like receptor
TREM-1: triggering receptor expressed on myeloid cells
RIG-I: retinoic acid inducible gene I
IFN-β: interferon beta
VES-13: vulnerable elders survey
qRT-PCR: quantitative reverse transcription polymerase chain reaction
LPS: lipopolysaccharide
