# Supplementary material for: Age, serum 25-hydroxyvitamin D and vitamin D receptor (VDR) expression and function in peripheral blood mononuclear cells
Source: Oncotarget. 2016 May 17;7(24):35512–21. doi: 10.18632/oncotarget.9398 (PMC5094941; doi:10.18632/oncotarget.9398)
Supplement: Supplementary file 1 [file oncotarget-07-35512-s001.pdf]

Age, serum 25-hydroxyvitamin D and vitamin D receptor (VDR) expression and function in peripheral blood mononuclear cells

Supplementary Material

Supplementary Materials: The following are available online at www.mdpi.com/link, Table S1: Gene-specific primers used in real-time PCR.

Table S1. Gene-specific primers used in real-time PCR

Target	PRIMER	Sequence 5′-3′
β-actin	Forward	ACCAACTGGGACGACATGGAGAAA
	Reverse	TAGCACAGCCTGGATAGCAACGTA
VDR	Forward	TGGCTTTCACTTCAATGCTATGA
	Reverse	CGTCGGTTGTCCTTGGTGAT
1,25D_3-MARRS	Forward	ATGGGCCTGTGAAGGTAGTG
	Reverse	GACCACACCAAGGGGCATAA
Cathelicidin	Forward	TGATAAGGATAACAAGAGATTTGCC
	Reverse	GTCCTGGGTACAAGATTCCG
TREM-1	Forward	CAGAACTGTGACCCAAGCTC
	Reverse	CGGAACCCTGATGATATCTGT
RIG-1	Forward	TGTGGGCAATGTCATCAAAA
	Reverse	GAAGCACTTGCTACCTCTTGC
1α-OHase	Forward	TGTTTGCATTTGCTCAGA
	Reverse	CCGGGAGAGCTCATACAG
IFN-β	Forward	CAACTTGCTTGGATTCCTACAAAG
	Reverse	TATTCAAGCCTCCCATTCAATTG
